# A Computational Framework for Automated Puncture Trajectory Planning in Hemorrhagic Stroke Surgery

**DOI:** 10.1002/brb3.70480

**Published:** 2025-04-21

**Authors:** Ziyue Ma, Feng Yan, Yongzhi Shan, Yaming Wang, Hong Wang

**Affiliations:** ^1^ Department of Neurosurgery Capital Medical University Xuanwu Hospital Beijing China; ^2^ Tianjin Key Laboratory of Neuromodulation and Neurorepair, Institute of Biomedical Engineering Chinese Academy of Medical Sciences & Peking Union Medical College Tianjin China

**Keywords:** hemorrhagic stroke, computational surgery, deep learning, path planning, geometric optimization

## Abstract

**Background:**

The treatment surgery for hemorrhagic stroke typically involves a puncture drainage procedure to remove the hematoma. However, the puncture targets for puncture and the puncture trajectory significantly influence the therapeutic outcome. This study proposes a computational framework integrating artificial intelligence (AI)‐driven segmentation, principal component analysis (PCA), and empirical optimization to automate puncture path generation.

**Methods:**

A software platform named Puncture Trajectory ToolKits (PTK) was developed using C++/Python with ITK/VTK libraries. Key innovations include hybrid segmentation that combines ResNet‐50 deep learning and adaptive thresholding for robust hematoma detection. PCA‐based longest axis extraction was enhanced by Laplacian mesh smoothing. Skull quadrant theory and safety corridor modeling were used to avoid critical structures. Five complex clinical cases were used to validate the framework's performance.

**Results:**

The framework demonstrated high accuracy in puncture trajectory planning, with the optimized L2 path achieving a mean surgeon satisfaction score of 4.4/5 (Likert scale) compared to manual methods. The average angle difference between automatically generated and manually designed paths was 16.36°. These results highlight PTK's potential to enhance the efficiency and safety of robotic‐assisted neurosurgery.

**Conclusion:**

PTK establishes a systematic pipeline for trajectory planning assistance, demonstrating technical superiority over conventional methods. The high acceptance rate among surgeons and improved planning efficiency underscore its clinical applicability. Future integration with robotic systems and validation through clinical trials are warranted.

## Graphical abstract

Neurosurgery Puncture Toolkit (PTK) generated paths with surgeon‐planned trajectories: red line L1 was the longest axis of hematoma as the first reference line, black point was the centroid of hematoma; blue line L2 was the second reference line as an optimized path. Yellow line was drawn manually as puncture path by the doctor.

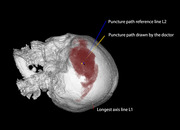

## 1 INTRODUCTION

Hemorrhagic stroke accounts for 15%–20% of stroke cases but contributes to over 40% of stroke‐related deaths (Feigin et al. 2017). Minimally invasive puncture surgery has become the gold standard for hematoma evacuation due to its reduced tissue damage (Walia et al. 2021). However, two critical challenges persist.

Anatomical complexity: The optimal trajectory must maximize hematoma coverage while avoiding eloquent areas (e.g., internal capsule, ventricles) (Vakharia and Duncan 2020). Manual planning introduces significant inter‐operator differences, with reported trajectory deviations exceeding 5 mm in 30% of cases (Boutet et al. 2019).

Surgical robots in neurosurgery are user‐friendly and highly efficient, increasingly becoming the preferred assistive devices in clinical treatments for cerebral hemorrhage. These devices leverage artificial intelligence to perform lesion identification and processing on medical images of patients, making the segmentation of cerebral hemorrhages and brain tumors among the hottest topics in academic research (Moccia et al. 2018). Surgical planning schemes lack substantial research due to their complexity involving clinical experience and various influencing factors. In clinical practice, numerous considerations influence the cerebral hemorrhage puncture trajectory, primarily concerning drainage effectiveness, blood vessels, and functional brain regions to prevent new hemorrhagic injuries, functional zone damage, and incomplete hematoma removal. Therefore, this study proposes an empirical automatic puncture trajectory algorithm based on lesion segmentation, aiming to provide intelligent reference trajectory during surgeons' surgical plan design.

## 2 Methods

### 2.1 Medical Images Case Selection

The experimental data used in this study were obtained from the publicly available datasets Kaggle (Intracranial hemorrhage detection dataset 2019), specifically designed for research purposes in the field of medical imaging. These datasets consist of computed tomography (CT) scans that simulate intracranial hemorrhage cases, providing a realistic yet non‐clinical source of data. Since the data are derived from public repositories rather than clinical sources, ethical approval was not required for their use. Clinical datasets will be used in future studies to enhance real‐world applicability.

### 2.2 Hematoma Segmentation

There have been numerous studies and reports on deep learning methods for brain CT image segmentation in intracranial hemorrhages (Chilamkurthy et al. 2018; Xu et al. 2024; Zhou et al. 2020), among which convolutional neural network (CNN). Segmentation of intracerebral hemorrhage regions is relatively efficient. We chose to reference the research by T. Lewick, and others are using a residual network (Res‐Net) model trained on the dataset to obtain a weight model for extracting the intracranial hematoma region from patient case images (Boutet et al. 2019; Lewick et al. 2020). The segmented region was then processed into a point cloud for subsequent calculations of the puncture centroid and longest axis.

In this study, preoperative imaging of patients with acute hemorrhage, characterized by a large volume of bleeding, early progression, and suitability for threshold segmentation methods, was directly segmented using a pixel threshold range of 60−80 Hu. The image of the main hemorrhagic area was obtained through processing methods such as maximum connected region analysis, which can then be further used to complete the planning and calculation of the puncture target and puncture route.

### 2.3 Calculation of Puncture Centroid and Longest Axis

The hematoma region extracted by the deep learning model was used to calculate the centroid (center of mass) of all points in the lesion area. Then, an inertia matrix was constructed using the lesion point cloud, which describes the object's resistance to rotation. Its eigenvectors point in the direction of the object's principal axes. By performing eigenvalue decomposition on the inertia matrix, three eigenvalues and their corresponding eigenvectors are obtained. The eigenvalues are sorted, and the corresponding eigenvectors represent the principal axis directions. The longest axis corresponds to the smallest eigenvalue since the minimum eigenvalue of the inertia matrix represents the least inertia when the object rotates along that direction. Another approach is to perform segmented region principal component analysis to find the longest path through the region. The location for needle insertion is provided by a fixed distance (usually 10−15cm) from the puncture target along the longest axis line L1 (C is the covariance matrix, and I is the inertia matrix; $\lambda_{i}$ is the ith eigenvalue, and $v_{i}$ is the corresponding eigenvector).

(1) $$C=\left(\begin{array}{ccc} \mathrm{Var}\left(X\right) & \mathrm{Cov}\left(X,Y\right) & \mathrm{Cov}\left(X,Z\right)\\ \mathrm{Cov}\left(Y,X\right) & \mathrm{Var}\left(Y\right) & \mathrm{Cov}\left(X,Z\right)\\ \mathrm{Cov}\left(Z,X\right) & \mathrm{Cov}\left(Z,Y\right) & \mathrm{Var}\left(Z\right)\; \end{array}\right),$$

(2) $$C_{vi}=\lambda_{i}\;v_{i};\;I_{vi}=\lambda_{i}\;v_{i}.$$

### 2.4 Reference Trajectory for Empirical Puncture Paths

#### 2.4.1 Coordinate System and Cerebral Quadrant Theory

We established a three‐dimensional Cartesian coordinate system (coronal, sagittal, horizontal planes) based on CT images, setting the center of the brain as the origin of the coordinate system, typically aligned with the center of the CT image or the central point of the ventricular system. The skull is divided into eight quadrants along the coronal and sagittal axes (four quadrants at the top of the head and four at the face/lower part), with the positive direction of the *z*‐axis being upward from the top of the cranium. For reference trajectory design, we draw a reference trajectory from the brain's center point toward the center of each of the four upper quadrants: The angle between these lines and each axis is 45°. The direction vectors are defined as follows: front‐upper quadrant:(1,1,1), back‐upper quadrant:(−1,1,1), front‐lower quadrant: (1,−1,1), and back‐lower quadrant:(−1,−1,1). These lines serve as directional references for puncture path planning.

#### 2.4.2 Puncture Path Planning

**FIGURE 1:**
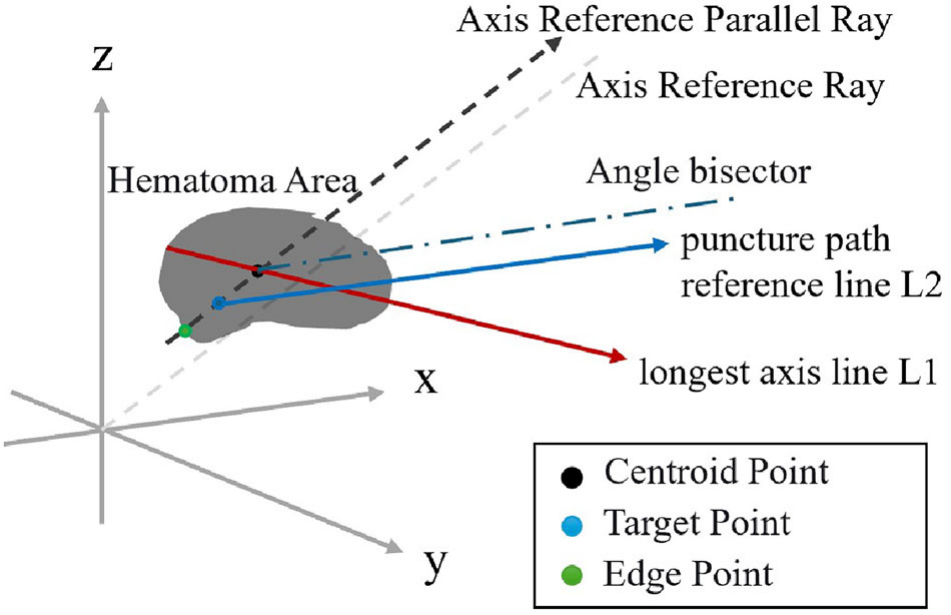
Schematic of skull quadrant partitioning and reference line directions for puncture trajectory planning.

Based on the position of the hematoma's centroid, the puncture path is planned as follows. For the upper part (z≥0): If the centroid is located in one of the upper quadrants (z≥0), the puncture path direction is parallel to the corresponding reference trajectory of that quadrant. The entry point is chosen as the point on the outer surface of the skull closest to the centroid, and the puncture target is the centroid itself. For the lower part (z<0): If the centroid is located in one of the lower quadrants (z<0), the planning still refers to the direction of the upper quadrant's reference trajectory. However, to avoid critical anatomical structures, the path is adjusted to bypass areas such as the nasal cavity and the base of the skull. The puncture path is designed as the bisector of the angle formed by the reference trajectory parallel to the quadrant containing the centroid and the longest axis passing through the centroid, defining the reference direction vector Vr. The reference target is further optimized and set as the midpoint between the intersection of the bisector line with the hematoma boundary and the centroid. Using this reference target and reference direction vector Vr, the second reference trajectory L2 is drawn. The illustration of the quadrant theory is shown in Figure 1.

Principal component analysis (PCA) identifies the hematoma's longest axis by decomposing its spatial covariance matrix. This axis represents the optimal geometric orientation for maximizing hematoma coverage during drainage. In skull quadrant theory, the skull is divided into eight quadrants based on anatomical landmarks (coronal/sagittal planes). Reference lines from the brain's center to quadrant midpoints guide trajectory directions, ensuring alignment with safe surgical corridors (e.g., avoiding the Sylvian fissure). PCA defines the hematoma's geometric axis, while quadrant theory constrains trajectories to anatomically safe directions. Their synergy ensures paths are both geometrically optimal and clinically feasible. This dual approach enhances drainage efficiency and operational safety compared to L1, which relies solely on the PCA axis. Clinical evaluations further validated L2's superiority, with surgeons rating it higher in feasibility.

### 2.5 Comparison of Manual Path and Two Reference Path

To evaluate the accuracy and effectiveness of the puncture path design, the paths planned for the included patient cases were compared with the automated reference paths. The analysis focused on two key aspects: the angle between the extended lines of the planned and reference paths, and the distance from the planned path's endpoint to the puncture target. This evaluation aimed to assess the discrepancies between the planned puncture paths and those actually used during clinical procedures.

### 2.6 Design Principles and Framework for Puncture Path Program

**FIGURE 2:**
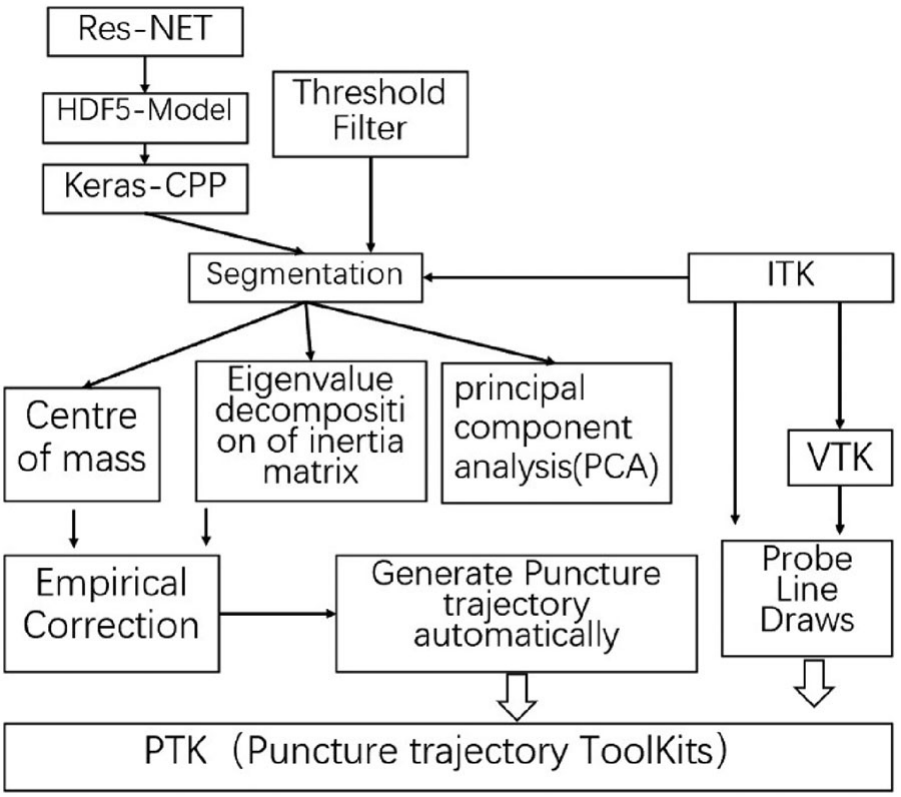
Architecture of the Puncture Trajectory Toolkit (PTK) computational framework.

As shown in Figure 2, puncture trajectory toolkits (PTK) is a C++‐based application designed for neurosurgical planning. The software leverages the Insight Segmentation and Registration Toolkit (ITK) for image processing and the Visualization Toolkit (VTK) for 3D rendering and display. The empirical correction model, described in Section 2.4, serves as the core algorithm module for optimizing puncture paths. PTK features an intuitive user interface, allowing surgeons to visualize and adjust trajectories in real time across multiple views (axial, sagittal, coronal, and 3D). Additionally, the software supports interactive target point selection and path comparison, enhancing its usability in clinical settings.

### 2.7 Statistical Analysis

Paired *t*‐tests were conducted to compare the distances between L1/L2 puncture targets and manually planned points, as well as the angular differences between automated and manual paths. A significance level of *α* = 0.05 was adopted, and *p*‐values were calculated using Python SciPy (version 1.10.0).

### 2.8 Clinical Relevance of the Algorithm

The longest axis (L1) ensures maximum hematoma coverage, while the optimized L2 path balances surgical safety by aligning with skull anatomy. The quadrant theory‐based design mimics neurosurgeons’ empirical planning patterns, making the algorithm intuitive for clinical adoption. However, puncture trajectory planning inherently involves subjective clinical judgment, as surgeons balance anatomical constraints and individual experience. To address this variability, we incorporated a clinical evaluation (1–5 scoring system) by three neurosurgeons to assess path feasibility, safety, and drainage efficiency. While angle differences provide quantitative insights, the scale captures multidimensional clinical acceptability, ensuring alignment with real‐world surgical decision‐making.

## 3 Results and Analysis

### 3.1 Development and Design of Puncture Planning Tool Software

**FIGURE 3:**
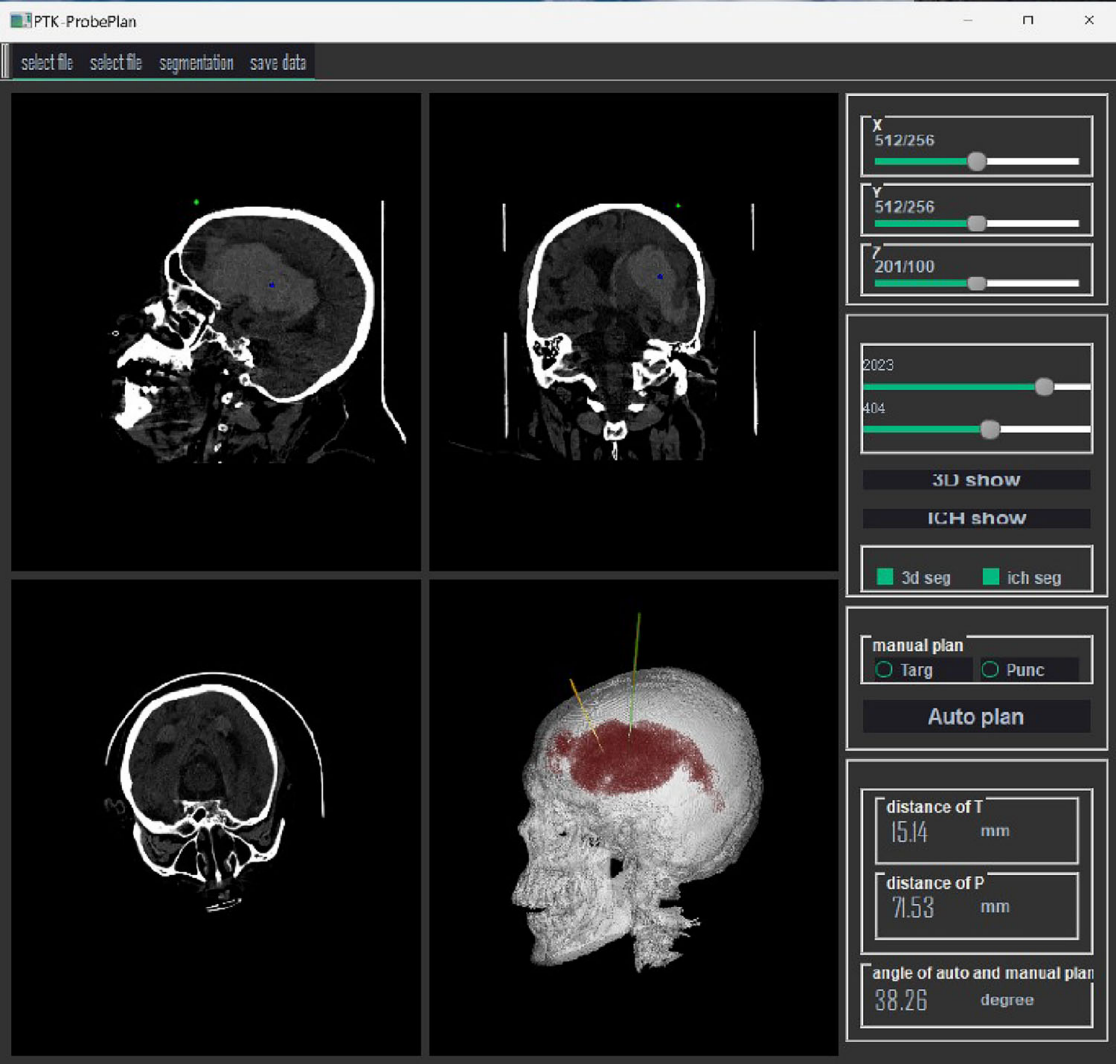
PTK software interface for hematoma segmentation and puncture path planning.

**FIGURE 4:**
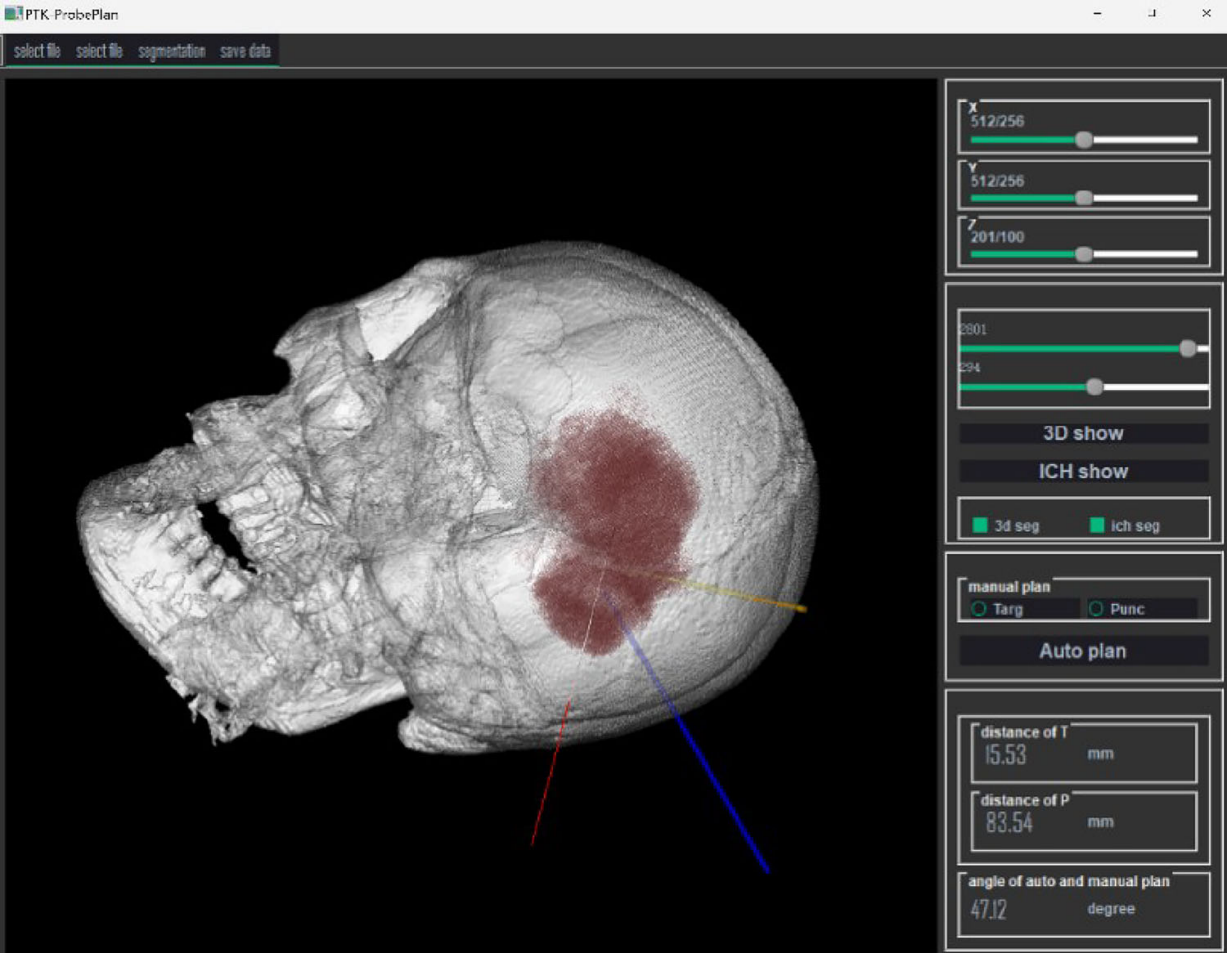
Comparison of PTK‐generated paths with surgeon‐planned trajectories (yellow line: manually planned; red line: first reference line main component centroid path; blue line: second reference line optimized path).

In the design of the puncture planning software, we prioritized practicality by creating a single‐page application. The main view includes coronal, axial, sagittal, and three‐dimensional perspectives. Each of these views can be double‐clicked to maximize into a single view, and another double‐click can restore the four‐panel layout. The left toolbar features functions such as threshold segmentation, hematoma segmentation rendering, manual annotation of puncture targets and points, automatic puncture path planning, and comparison of manual and two automatic path results. The software operator interfaces are illustrated in Figures 3 and 4.

### 3.2 Intracranial Hematoma Image Segmentation

In this study, preoperative CT images of 10 cases with acute intracranial hemorrhage were analyzed. We used deep learning methods and threshold segmentation (pixel values between 60 and 80) to segment the hematoma. The volumes of the segmented hematomas were calculated, and the results shown in Table 1 from the two methods were compared. First, using the deep learning method, we obtained an average volume difference between the two segmentation methods. Statistical testing (*t*‐test) showed no significant difference between them (p>0.05). This indicates that although deep learning methods may have higher flexibility and accuracy in dealing with complex images, their segmentation performance is comparable to traditional threshold methods in our selected standard samples of acute intracranial hemorrhage. Both methods performed well in capturing the boundaries of the hematoma and preserving the morphological features of the hematoma. Combining the advantages and disadvantages of both methods, we believe that in clinical practice, the segmentation method can be flexibly chosen based on specific application scenarios.

**TABLE 1:** Comparison of hematoma segmentation accuracy between artificial intelligence (AI) and threshold‐based methods.

Index	1	2	3	4	5
Volume of AI segmentation (mm^3^)	90.5	80.0	80.2	100.0	75.0
Volume of threshold filter segmentation (mm^3^)	85.0	83.5	82.0	98.0	70.0
Variation (mm^3^)	5.5	−3.5	−1.8	2.0	5.0

### 3.3 Comparison of Clinical Puncture Targets Data

We performed a segmentation analysis of intracranial hemorrhage for 10 cases, comparing deep learning methods with traditional threshold segmentation (pixel values 60−80). In Table 2, we calculated the distance between the centroid point (puncture target) of the segmented image and the puncture target planned by the clinician. Although the *p*‐value (*p* = 0.12) did not reach statistical significance, the mean distance difference (16.98 mm) falls within the clinically acceptable range for minimally invasive procedures. Compared the target's difference between the automatically generated puncture route (longest axis) and the puncture route designed by the doctor. The detailed data and analysis results for each patient are as follows.

**TABLE 2:** Distance deviations between automated reference path targets (L1, L2) and surgeon‐planned targets.

Index	1	2	3	4	5
Target's distance of L1 to manual Line (mm)	8.31	15.52	10.02	27.5	23.54
Target's distance of L2 to manual Line (mm)	5.20	7.03	3.54	13.55	15.53

### 3.4 Angle Difference in Puncture Route

The angle difference between the automatically generated puncture route L2 and the doctor's designed puncture route showed that L2 is closer to the actual puncture path of the doctor compared to L1 (*p* <0.05). The specific data are shown in Table 3. Automatically Generated Puncture Route vs. Doctor's Designed Puncture Route.

**TABLE 3:** Automatically generated puncture route versus doctor's designed puncture route angle.

Index	1	2	3	4	5
Angle of L1 and manual line(°)	40.28	60.02	116.78	43.36	76.45
Angel of L2 and manual line(°)	5.50	13.33	10.67	5.20	47.12

### 3.5 Clinical Acceptance Criteria and Surgeon Feedback

While direct clinical outcome data (e.g., postoperative complications, recovery time) are beyond the scope of this computational study, the software's clinical utility was preliminarily validated through a surgeon‐centered evaluation. Three neurosurgeons with >10 years of experience in hemorrhagic stroke interventions were invited to assess the PTK‐generated paths using a standardized questionnaire (Table 4). Key metrics included:

**TABLE 4:** Surgeon evaluation of PTK‐generated paths (*n* = 3).

Index	L1 path score (Mean ± SD)	L2 path score (Mean ± SD)
Safety (1–5)	3.2 ± 0.8	4.4 ± 0.5
Drainage efficiency (%)	68 ± 12	85 ± 8

Path safety: Likert scale (1–5) rating of whether the trajectory avoided critical structures.

Operational feasibility: Time required to finalize the path (manual vs. PTK‐assisted).

Drainage efficiency: Subjective assessment of hematoma coverage based on 3D visualization.

The results showed a mean satisfaction score of 4.4/5 for L2 paths. Although this evaluation does not replace rigorous clinical trials, it aligns with the United States Food and Drug Administration's guidance on early feasibility assessments for surgical planning tools, which emphasize workflow integration and expert acceptance as critical benchmarks.

## 4 Discussion

Based on the above data analysis, the average distance between the centroid point and the doctor's planned puncture target is about 17mm, demonstrating the effectiveness of the segmentation algorithm in puncture target localization. Although the distance difference between the centroid point and the doctor's puncture target did not reach significance, it is within an acceptable range in clinical applications, especially in minimally invasive surgery where precise puncture target localization is crucial. Relevant literature has shown that the effectiveness of centroid points as targets has been validated in multiple studies (Calusi et al. 2021, Vakharia and Duncan 2020). These studies support the potential of centroid points as surgical targets, especially when dealing with complex hematoma.

In terms of puncture path design, the average angle difference between the automatically generated puncture route and the doctor's designed puncture route showed that was L1 line was closer with actually doctor than L2 line, demonstrating the high reliability of the algorithm in puncture path planning. This result is consistent with current literature on automated path planning, which shows that image segmentation‐based automation methods can significantly improve puncture path accuracy and reduce surgical risks (Walia et al. 2021). This consistency not only enhances the credibility of the algorithm but also provides a solid foundation for clinical practice, indicating the potential of intelligent surgical planning in improving surgical safety and effectiveness.

The quadrant reference trajectory designed in this study is guided by the convenience of actual surgical operations and puncture angles. It introduces the influence of the longest axis based on the parallel lines of the quadrant reference rays. The puncture path is designed as the bisector of the angle formed by the reference trajectory parallel and the longest axis passing through the centroid point, thereby addressing the issue of the smallest tangent angle between the longest axis and the skull (i.e., insufficient path inclination, which may affect puncture effectiveness). It improves hematoma drainage efficiency, making the path more conformal to the geometric shape of the hematoma.

The new puncture target for the second reference trajectory is adjusted to the midpoint between the intersection point where the bisector passes through the centroid toward the hematoma boundary and the centroid point itself. The intersection point is where the extended bisector meets the boundary of the hematoma region, and the midpoint is the central location from the centroid to the intersection point. The position of the puncture target leans toward the lower part of the hematoma, aiding in a more comprehensive drainage of hematoma contents. The average distance between the puncture target point of L2 and the doctor's planned puncture target is about 9 mm which seems closer than centroid point.

The rationality of the second reference trajectory path lies in its design that combines the longest axis and skull structure, avoiding situations where the path has too small a tangent with the skull surface, reducing surgical difficulty and risk of complications. At the same time, it can improve hematoma drainage efficiency and enhance postoperative outcomes. Additionally, the puncture target position has been optimized, with the new target selection leaning more toward the lower part of the hematoma, aligning with the goal of draining more hematoma content. The midpoint strategy balances the stability of puncture target positioning and the feasibility of clinical operations. This theory has universal applicability, suitable for different types of cranial hematomas, especially those with complex geometric shapes or wide distribution ranges. It provides a new mathematical model and path planning method, which can be further applied to the implementation of robotic navigation systems.

On the other hand, we also recognize certain limitations of this design. Due to the complexity of hematoma distribution, the theoretical assumption that the shape and principal axis direction can well reflect the distribution trend might not always hold true in practice. Hematomas may present irregular shapes, and the principal axis may not fully cover critical areas. The bisector may deviate from the actual optimal path, requiring subsequent optimization verification. More clinical validations are needed, particularly in terms of safety and operational feasibility, through comparisons with paths planned by physicians.

The puncture path theory, by incorporating reference rays, the longest axis, and the bisector method, forms a more scientific framework for puncture path design. The adjustment of puncture targets also reflects a design philosophy oriented toward clinical needs, optimizing drainage effects while enhancing the rationality and feasibility of path planning. This method is suitable for application in image‐segmentation‐based automated path planning systems and provides theoretical support and design ideas for robot‐assisted cranial hematoma puncture.

The current algorithm assumes hematomas are convex‐shaped. The algorithm includes Laplacian mesh smoothing to handle mild irregularities. For multilobed cases, future versions will incorporate multi‐axis PCA or graph‐based partitioning. Irregular hematomas (e.g., septated or multi‐lobed) may require adaptive segmentation methods. Additionally, the validation was limited to simulated data; future studies should incorporate intraoperative navigation and postoperative outcome tracking. Future work can validate the effectiveness of this new theory through clinical data and further optimize path planning algorithms for complex hematoma shapes. Inter‐operator variability was not assessed in this pilot study. Future work will involve multiple surgeons to quantify variability and compare it with AI consistency. A prospective clinical trial is planned. Integration with multimodal imaging (e.g., CTA for vascular mapping) and real‐time robotic navigation will be prioritized. Machine learning–based shape adaptation algorithms are under development to handle complex hematoma geometries.

## 5 Conclusion

The PTK framework automates puncture path planning for hemorrhagic stroke by integrating AI segmentation, PCA analysis, and clinical expertise. Case studies demonstrate L2's superiority in perpendicularity, drainage efficiency, and operational time. This tool provides robust theoretical and software support for robot‐assisted surgery, with promising potential for clinical translation.

## Author Contributions

**Ziyue Ma**: Writing–original draft; software. **Yaming Wang**: Validation. **Hong Wang**: Conceptualization; investigation; project administration; funding acquisition. **Feng Yan**: Validation; investigation; writing–review and editing. **Yongzhi Shan**: Validation; supervision.

### Peer Review

The peer review history for this article is available at https://publons.com/publon/10.1002/brb3.70480

## Data Availability

The data that support the findings of this study are openly available in intracranial hemorrhage detection dataset at https://www.kaggle.com/c/rsna‐intracranial‐hemorrhage‐detection/data.

## References

[brb370480-bib-0001] Boutet, A. , R. Gramer , C. J. Steele , et al. 2019. “Neuroimaging Technological Advancements for Targeting in Functional Neurosurgery.” Current Neurology and Neuroscience Reports 19: 42. 10.1007/s11910-019-0961-8. 31144155

[brb370480-bib-0002] Calusi, S. , C. Arilli , E. Mussi , et al. 2021. “In Phantom Evaluation of Targeting Accuracy in MRI‐Based Brain Radiosurgery.” Physica Medica 85: 158–164. 10.1016/j.ejmp.2021.05.014. 34015617

[brb370480-bib-0003] Chilamkurthy, S. , R. Ghosh , S. Tanamala , et al. 2018. “Deep Learning Algorithms for Detection of Critical Findings in Head Ct Scans: A Retrospective Study.” The Lancet 392: 2388–2396. 10.1016/S0140-6736(18)31645-3. 30318264

[brb370480-bib-0004] Feigin, V. L. , B. Norrving , and G. A. Mensah . 2017. “Global Burden of Stroke.” Circulation Research 120: 439–448. 10.1161/CIRCRESAHA.116.308413. 28154096

[brb370480-bib-0005] Intracranial hemorrhage detection dataset . 2019. “RSNA Intracranial Hemorrhage Detection.” https://www.kaggle.com/c/rsna‐intracranial‐hemorrhage‐detection/data.

[brb370480-bib-0006] Lewick, T. , M. Kumar , R. Hong , and W. Wu . 2020. “Intracranial Hemorrhage Detection in Ct Scans Using Deep Learning.” Paper presented at the 2020 IEEE Sixth International Conference on Big Data Computing Service and Applications (BigDataService), Oxford, UK. 10.1109/BigDataService49289.2020.00033.

[brb370480-bib-0007] Moccia, S. , E. De Momi , S. El Hadji , and L. S. Mattos . 2018. “Blood Vessel Segmentation Algorithms—Review of Methods, Datasets and Evaluation Metrics.” Computer Methods and Programs in Biomedicine 158: 71–91. 10.1016/j.cmpb.2018.02.001. 29544791

[brb370480-bib-0008] Vakharia, V. N. , and J. S. Duncan . 2020. “Automation Advances in Stereoelectroencephalography Planning.” Neurosurgery Clinics 31: 407–419. 10.1016/j.nec.2020.03.005. 32475489 PMC7902942

[brb370480-bib-0009] Walia, S. , K. Fisher , R. L. Dodd , and C. Venkatasubramanian . 2021. “Surgical Management of Spontaneous Intracerebral Hemorrhage.” Current Treatment Options in Neurology 23: 28. 10.1007/s11940-021-00678-0.

[brb370480-bib-0010] Xu, W. , Z. Sha , T. Tan , et al. 2024. “Automatic Segmentation of Intracranial Hemorrhage in Computed Tomography Scans With Convolution Neural Networks.” Journal of Medical and Biological Engineering 44: 575–581. 10.1007/s40846-024-00892-6.

[brb370480-bib-0011] Zhou, Z. , M. M. R. Siddiquee , N. Tajbakhsh , and J. Liang . 2020. “Unet++: Redesigning Skip Connections to Exploit Multiscale Features in Image Segmentation.” IEEE Transactions on Medical Imaging 39: 1856–1867. 10.1109/TMI.2019.2959609. 31841402 PMC7357299

